# Gender Specific Reproductive Strategies of an Arctic Key Species (*Boreogadus saida*) and Implications of Climate Change

**DOI:** 10.1371/journal.pone.0098452

**Published:** 2014-05-28

**Authors:** Jasmine Nahrgang, Øystein Varpe, Ekaterina Korshunova, Svetlana Murzina, Ingeborg G. Hallanger, Ireen Vieweg, Jørgen Berge

**Affiliations:** 1 Department of Arctic and Marine Biology, UiT The Arctic University of Norway, Tromsø, Norway; 2 University Centre in Svalbard, Longyearbyen, Norway; 3 Akvaplan-niva, Fram Centre, Tromsø, Norway; 4 Institute of Biology of the Karelian Research Centre, Russian Academy of Science, Petrozavodsk, Russia; Technical University of Denmark, Denmark

## Abstract

The Arctic climate is changing at an unprecedented rate. What consequences this may have on the Arctic marine ecosystem depends to a large degree on how its species will respond both directly to elevated temperatures and more indirectly through ecological interactions. But despite an alarming recent warming of the Arctic with accompanying sea ice loss, reports evaluating ecological impacts of climate change in the Arctic remain sparse. Here, based upon a large-scale field study, we present basic new knowledge regarding the life history traits for one of the most important species in the entire Arctic, the polar cod (*Boreogadus saida*). Furthermore, by comparing regions of contrasting climatic influence (domains), we present evidence as to how its growth and reproductive success is impaired in the warmer of the two domains. As the future Arctic is predicted to resemble today's Atlantic domains, we forecast changes in growth and life history characteristics of polar cod that will lead to alteration of its role as an Arctic keystone species. This will in turn affect community dynamics and energy transfer in the entire Arctic food chain.

## Introduction

Climate variability and global warming have changed the Arctic, most notably seen in the abrupt decline in Arctic sea ice extent and thickness [1]. As the temperature is predicted to rise disproportionally more in the Arctic compared to global trends [2], these current and future perspectives are likely to pose a significant challenge for both the marine [3]–[5] as well as the terrestrial [6] Arctic ecosystems. Altered marine community structures [7]–[9] and a northward expansion of boreal species [10], [11] are common expectations, to some extent also supported by observations [12]. When species expand their distribution ranges northwards, it may lead to either co-existence with, or replacement of their native Arctic counterparts [13]. Mechanisms involved in such range expansions are often directly associated with changes in water masses [14], [15], but may also be more indirect through biological interactions [3], [8]. Commonly predicted replacements include the high Arctic calanoid *Calanus glacialis* being replaced by its more boreal sister taxa, *Calanus finmarchicus* [16], [17], as well as the replacement of the polar bear (*Ursus maritimus*) by killer whale (*Orcinus orca*) as a likely effect of increased temperatures and loss of sea ice in the Arctic [18].

In high Arctic marine ecosystems, the polar cod (*Boreogadus saida*) is regarded as a key link in the food web between lower and higher trophic levels [19], [20]. Polar cod has been a focal point for studies of climate [11], [21]–[23] and pollution impacts [24], [25]. Yet, current knowledge regarding this important Arctic species is surprisingly scattered and inconclusive, preventing a holistic understanding of its life history and ecology. Understanding life history strategies, the schedules of growth, fecundity and mortality [26], is imperative for our ability to predict species' responses to environmental change. Importantly, there are tight couplings among altered energy acquisition, survival, and consequently responses in energy allocation [27]. We need to cover these dimensions to understand how polar cod will respond to environmental changes.

**Figure 1:**
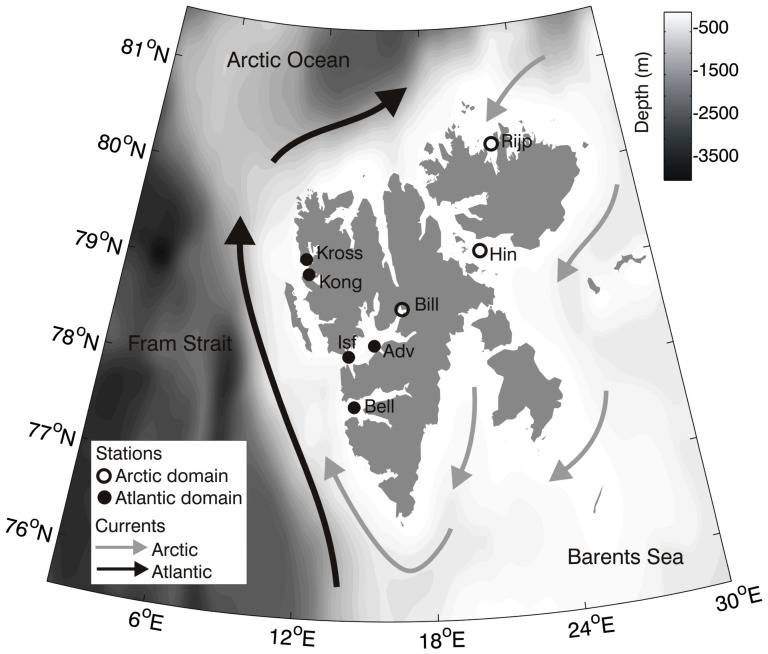
Sampling stations of Arctic and Atlantic domains. Map of the sampling stations with dominant current systems (Bell: Bellsund, Isf: Isfjorden, Adv: Adventfjorden, Bill: Billefjorden, Kong: Kongsfjorden, Kross: Krossfjorden, Hin: Hinlopen, Rijp: Rijpfjorden).

Through a large field study of wild polar cod populations from comparable shelf areas influenced by either Arctic or Atlantic water masses, hereafter called domains (Figure 1, Table S1), we established a natural large-scale climate experiment. We use this design to fill important gaps in knowledge regarding life history traits and reproductive strategies, and to test if they are stable across climatic domains and genders. Specifically, we ask if population structure, size-at-age, age-at-maturation, and reproductive strategy (including semelparity versus iteroparity) are similar across the two domains. Furthermore, we discuss potential direct and cascading ecological effects from a continued warming of the Arctic Ocean for arguably the most abundant and ecologically important fish species within the high Arctic food chain.

## Material and Methods

### Ethic statement

All work were performed according to and within the regulations enforced by the Norwegian Animal welfare authorities and no specific permissions were required. The R/V *Helmer Hanssen* is owned by the University of Tromsø and has all necessary autorization from the Norwegian Fisheries Directorate to use a bottom trawl to collect fish for scientific purposes. The lead author had all necessary training and certificates (FELASA C) to perform the work including sacrificing the sampled organisms. Furthermore, the organisms are neither protected nor endangered in the coastal waters of the Svalbard Archipelago. Upon trawling, polar cod were sacrificed by a sharp blow to the head and immediately dissected as specified below.

### Study sites and polar cod sampling

Polar cod were collected using a Campelen bottom trawl from R/V *Helmer Hanssen* between 2010 and 2013 from 8 stations (Figure 1, see also Table S1 for a detailed overview). Trawling time was standardised to 15 min bottom time for all trawl hauls used in population estimates (Table 1), but reduced to 5 min in Adventfjorden. The opening of the trawl is 60 m across, the trawling speed was set to 2 kt. In order to make the catches comparable, trawling was carried out at a depth between 180 and 220 m bottom (with one exception in Bellsund, 130 m), depending on the stations and on comparable bottom types. For the samples taken in January 2012 (Rijpforden and Isfjorden) and in January and September 2013 (Rijpfjorden, Isfjorden, Krossfjorden and Kongsfjorden), 100% of the trawl catches (all species and specimens included) were considered. For all other stations (Table S1), only random sub-samples of polar cod were treated. Also, due to the failure to adequately collect pelagic early life stages with the bottom trawl [11], larvae and juvenile fish of total length <10 cm were removed from subsequent analyses.

**Table 1:** Fish composition of benthic trawls taken in January 2012 and January and September 2013 in fjords from the Arctic and Atlantic domains.

	Arctic				Atlantic				
	Rijp	Rijp	Rijp	Hin	Adv	Isf	Kross	Isf	Kong
Taxon	Jan 2012	Jan 2013	Sept 2013	Sept 2013	Jan 2012	Jan 2013	Jan 2013	Sept 2013	Sept 2013
*Boreogadus saida*	***12.6 (1023)***	***14.4 (689)***	***22.3 (1325)***	***8.4 (310)***	***2.4 (306)***	0.6 (958)	1.1 (310)	23.4 (2358)	23.7 (2008)
*Gadus morhua*	5.5 (22)	0.4 (30)		0.07 (7)	***3.0 (300)***	***5.5 (89)***	***13.6 (163)***	***54 (22)***	***26.5 (7)***
*Mallotus villosus*	0.1 (6)	0.1 (45)	0.1 (5)	0.1 (7)	0.03 (3)	0.03 (7)	0.03 (2)		0.03 (3)
*Leptoclinus maculatus*	0.1 (6)	0.1 (28)	0.001 (1)	0.05 (10)	0.03 (3)	0.1 (26)		0.1 (19)	0.03 (5)
*Sebastes sp*		0.6 (138)	0.03 (4)	0.9 (16)	0.03 (6)	0.7 (26)	1.1 (51)	0.2 (1)	0.5 (7)
*Hippoglossoides platessoides*	1.3 (4)	0.3 (3)		0.8 (5)		3.8 (43)	0.1 (5)	0.8 (5)	2.4 (13)
*Reinhardtius hippoglossoides*	0.8 (1)			0.1 (5)	0.03 (9)	***5.3 (1)***	0.1 (1)	14 (24)	3.1 (7)
*Liparis sp*		0.3 (47)	0.4 (22)	0.02 (8)		0.04 (13)	0.1 (6)		0.02 (2)
*Leptagonus decagonus*		0.1 (2)		0.001 (1)		0.03 (4)	0.03 (6)		
*Melanogrammus aeglefinus*						0.1 (1)	1.6 (1)		0.01 (1)
*Triglops spp*		0.1 (23)		2.0 (2)					
*Eumicrotremus spinosus*		0.1 (3)		0.04 (4)					
*Clupea harengus*						0.5 (4)	0.01 (1)		
*Lycodes spp*						0.1 (4)		0.06 (1)	
*Myxocephalus sp*		0.1 (8)							

Total weight of each taxon (kg) and number of individuals in brackets. Dominant taxa according to biomass (>30% of total biomass, kg) in bold and italics. Trawl duration differed between stations, hence all numbers are standardized as catch per 15 min trawling time.

**Figure 2:**
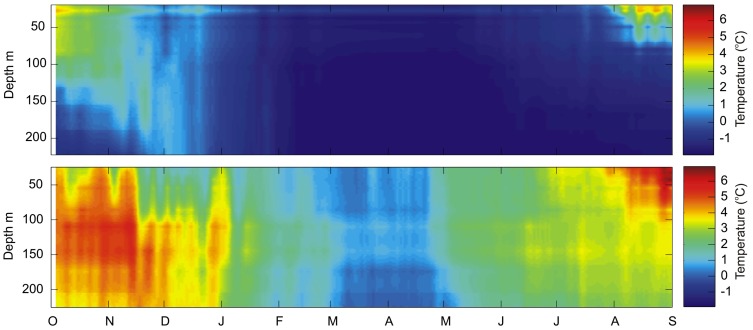
Seasonal temperature plots in an “Atlantic” and “Arctic” type fjord. Temperature plots from moored observatories between 4^th^ of October 2012 and 1^st^ of September 2013 in Rijpfjorden (upper) and Kongsfjorden (lower).

Oceanographic data (Figure 2) were gathered from two moorings deployed in Kongsfjorden and Rijpfjorden, respectively (see [28] for details). The moorings were located in the immediate vicinity (1 km apart) to the trawling grounds in both fjords, and have been in operation since 2002 as part of a long term monitoring program.

The polar cod total length (TL, ±0.1 cm) and/or fork length (FL, ±0.1 cm), total wet weight (TW, ±0.1 g wet weight) and sex were recorded for all collected individuals. TW was recorded for a large fraction of the individuals (n = 1124, Figure S1). Somatic weight (0.1 g wwt), gonad and liver weight (±0.01 g wwt) were recorded when possible in order to calculate the gonado-somatic (GSI, n = 314, Table 2) and hepato-somatic index (HSI, n = 266, Table S2) respectively. Otoliths were collected for age determination from the dissected subsamples. In addition, otoliths were collected non-randomly to cover all size ranges (n = 296). Stomachs were dissected out from a subsample of specimens collected in Rijpfjorden and Kongsfjorden in September 2013 (Table S1) and the fresh material was examined under a dissecting microscope. Identification of prey items was made to the lowest possible taxon based on degree of digestion. Results are presented as frequency of occurrence (Table 3).

**Table 2:** Description of the maturing fraction of polar cod.

	Arctic			Atlantic		
TL (cm)	n	Female	Male	n	Female	Male
**Percentage maturing**						
]9–12]	26	14	83	38	41	65
]12–15]	106	81	95	49	58	73
]15–18]	53	97	100	2	100	100
]18–21]	23	100	100			
**Gonado-somatic index of mature specimens**						
]9–12]		17.7±7.9^a^	22.1±2.8^a^		14.7±1.8	21.4±5.5
]12–15]		20.4±3.9^b^	26.2±7.2^a,b^		17.0±5.3	20.9±4.5
]15–18]		20.6±4.6^b^	28.2±4.6^b,c^		23.0	25.0
]18–21]		25.5±5.1^c^	34.4±6.8^c^			
**Estimation of amount eggs per female**						
]9–12]		7738			5555	
]12–15]		11813			9220	
]15–18]		19335			24381	
]18–21]		36530				

Percentage maturing individuals (GSI>10%), their gonado-somatic index (mean ±SD) and the estimated number of eggs per female per size class (total length, cm) from the Arctic (n = 207) and Atlantic (n = 107) domains, combined for January 2011, 2012, and 2013. Different letters (a, b, c) represent significant differences (Welch-ANOVA and Dunnett's T3) in GSI between size classes for each sex and domain.

**Table 3:** Frequency of occurrence (%) of preys in polar cod stomachs.

	Arctic	Atlantic
n examined	298	157
n with content	287	132
Frequency of occurence of prey		
Calanoidae	5%	48%
*Themisto libellula*	100%	10%
*Themisto abyssorum*	0%	25%
Krill	1%	13%
Mysids	1%	13%
Fish	0.3%	5%
Polychaeta	0%	2%
Amphipoda	2%	5%
Cumacea	0%	14%
*Pandalus borealis*	1%	12%

Stomach contents of polar cod sampled in September 2013, from the Arctic (Rijpfjorden) and Atlantic (Kongsfjorden) domains. Only stomachs with content were included in the calculations.

**Figure 3:**
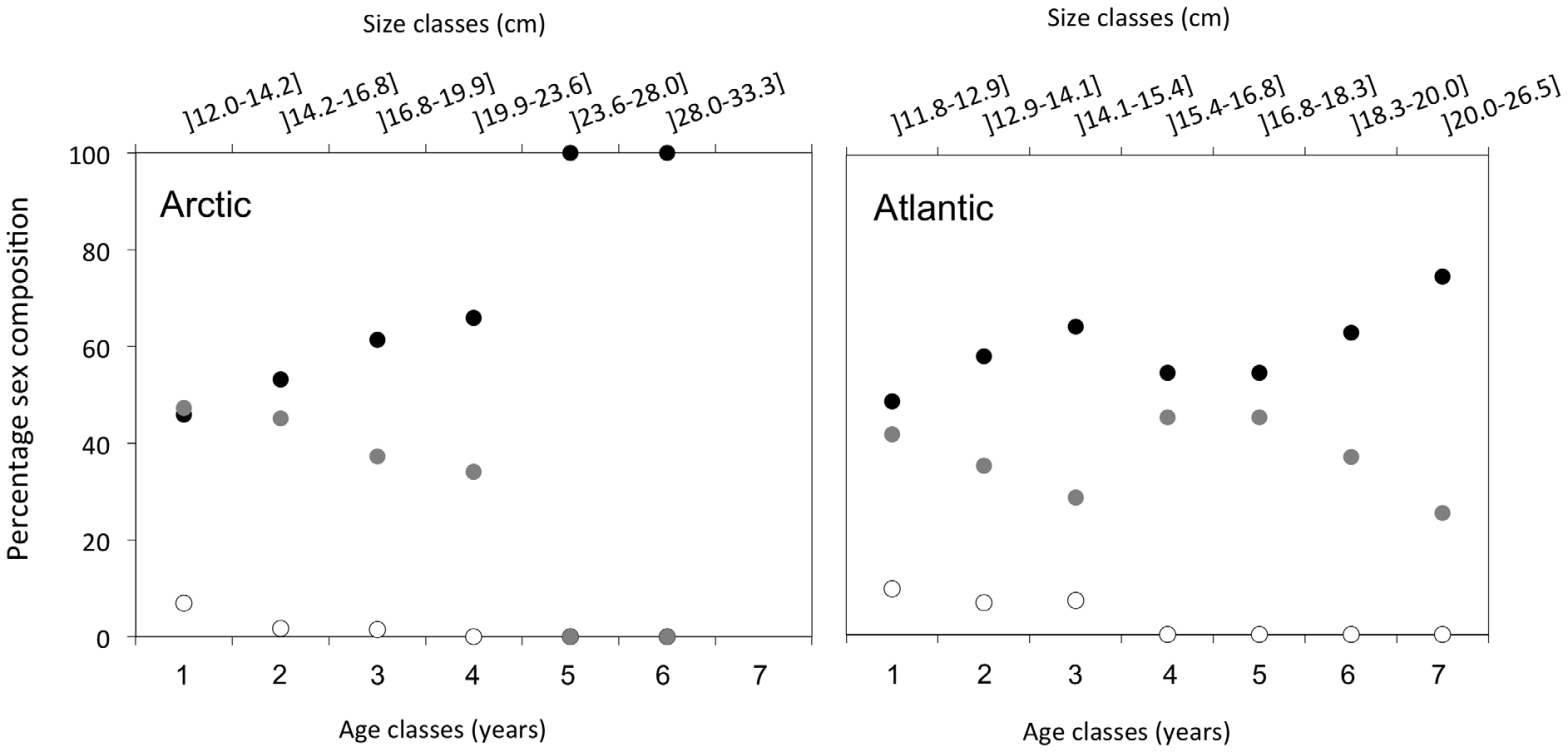
Sex ratio composition of polar cod in Arctic and Atlantic domains. Percentage female (black circles), male (grey circles) and immature (open circles) per age class of polar cod (lower x-axis) and size classes (cm, upper x-axis) from the Arctic (n = 1813) and Atlantic (n = 728) domains. Immature are individuals where gonads were not detectable. Age classes are derived from the linear regression presented in Figure 4, hence length ranges per age class differ for the two domains. Within the Arctic domain, a total of 74 females (max length 30.5 cm) and 30 males (max length 22.9 cm) were recorded larger than 20 cm. In the Atlantic domain only 53 females (max 26.5 cm) and 18 males (max length 25.0 cm) were recorded larger than 20 cm. For more details see Table S4.

All presented data are based on total length measurements. When only fork length (FL) was available, TL was determined with help of a linear regression FL = 0.9641 TL+0.0113 (R^2^ = 0.996), based on 1463 TL and FL records (see Figure S2). The GSI was calculated using the equation GSI = 100× gonad weight/somatic weight. Gonadal maturation was only considered from January samples, as males are known to increase gonad size several months before females [46], making direct comparison of GSI between genders impossible except in the pre-spawning period in January. As a threshold level for maturity, mature polar cod were defined by GSI>10% (Table 2). For the sex-ratio distributions (Figure 3), the category “immature” were specimens with unidentifiable gonads.

### Age estimation

**Figure 4:**
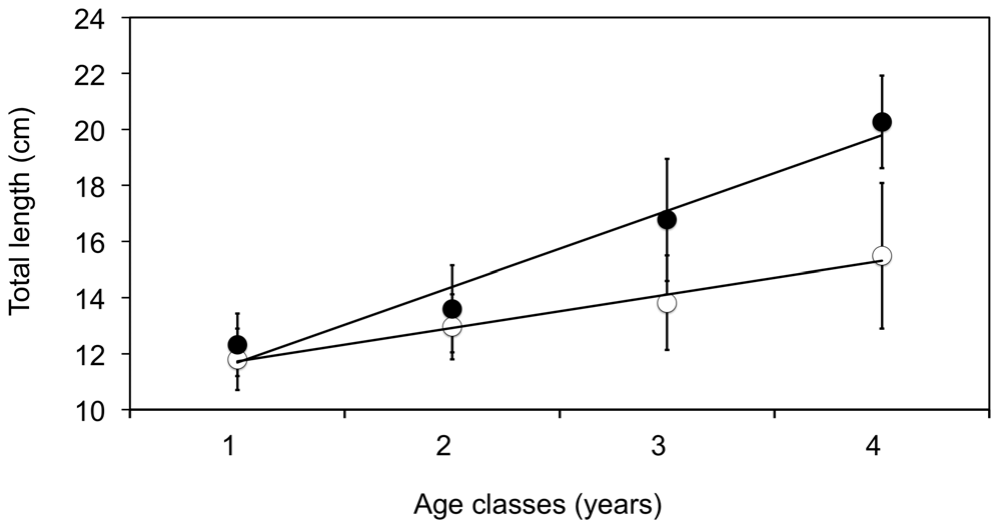
Relationship between total length (cm) and age (otolith based, years) of polar cod. Specimens from the Arctic (n = 97, closed) and Atlantic (n = 199, open) domains. Points indicate mean and wiskers are standard deviation, lines are predicted from a generalized linear model for the two domains where age was treated as a continuous variable. Arctic: TL = 2.710 Age+8.898 (R^2^ = 0.96) and Atlantic: TL = 1.0825 Age+10.756 (R^2^ = 0.98).

Polar cod age (years) was determined based on otolith readings: for small transparent otoliths, white winter rings were counted in sub-surface light with a Leica M205 C stereo microscope and a Planapo 1.0× objective lens; for all larger otoliths cross sectioning and counting the rings under polarised light was necessary. For individuals from which otoliths were dissected, age was estimated using a length-age relationships (Figure 4). Length (and not weight) was considered the most reliable proxy for age, as total weight is highly depending on maturation and feeding status of each individual fish.

### Histological analysis of gonads and estimation of fecundity

Gonads of mature females from January 2011 (n = 5) and January 2012 (n = 4) were analysed histologically to determine oocyte stages, size (diameter and area), and density represented as the percentage of picture area covered by oocytes related to total picture area within the gonads (Table S3). Classical histological methods using hematoxylin and eosin stains were used. Histological sections were studied in the light microscope (50–1000×) Axioskop 40 (Carl Zeiss) with eye lens ×10 and objective lenses ×5, ×10, ×20, ×40, ×100. Sections on the slides were photographed (Pixera Pro 150ES) and analysed with the Videotest programme. No significant differences in oocyte size and developmental stage were found between domains, suggesting that polar cod from both domains were in the same stage of oocyte development. Oocytes in the vitellogenesis stage represented 98.8% of the weight percentage of all oocyte stages, hence fecundity calculations were based solely on this dominating stage (Table S3). Vitellogenic oocytes represented 49±19% (n = 24 slices) of the total volume of the gonads. Fecundity (amount of egg) was then estimated based on the proportion of maturing females within size classes in the January 2011, 2012 and 2013 populations and their gonad weight (Table 2). Population fecundity was estimated based on the trawl catches (Figure S3) representing 5 examples of populations in the Arctic and Atlantic domains (see Table 1). The amount of maturing females per size class (*N_f_mat_*) in these populations was calculated, using the proportion of females-at-size data (Figure 3) and the proportion of maturing females-at-size data (Table 2) for each domain. The total amount of eggs (Σ*_Neggs_*) produced in the entire populations was then estimated based on the number of eggs (*N_eggs_*) produced per maturing female-at-size. *N_eggs_* assumes that 100% of the vitellogenic oocytes will mature into eggs. Vitellogenic oocyte weight (g) was estimated assuming neutral buoyancy: 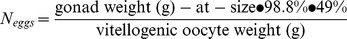


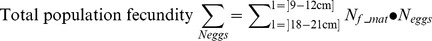


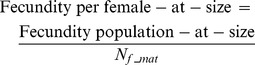



### Statistical analyses

A linear model (LM) was used to predict body length based on age, gender (male and female) and domain (factor with the two levels “Arctic” and “Atlantic”, Table S4). Age was treated as a continuous variable. 281 individuals could enter this analyses, that is fish longer than 10 cm in length and younger than 5 years of age (Table S4). For the LM we started out with a full model, including all interactions, and performed model simplification by stepwise removal of non-significant terms as suggested by Crawley [29]. Abundance and HSI were not included as potential explanatory variables due to lack of sufficient data. The LM was performed in R [29].

**Figure 5:**
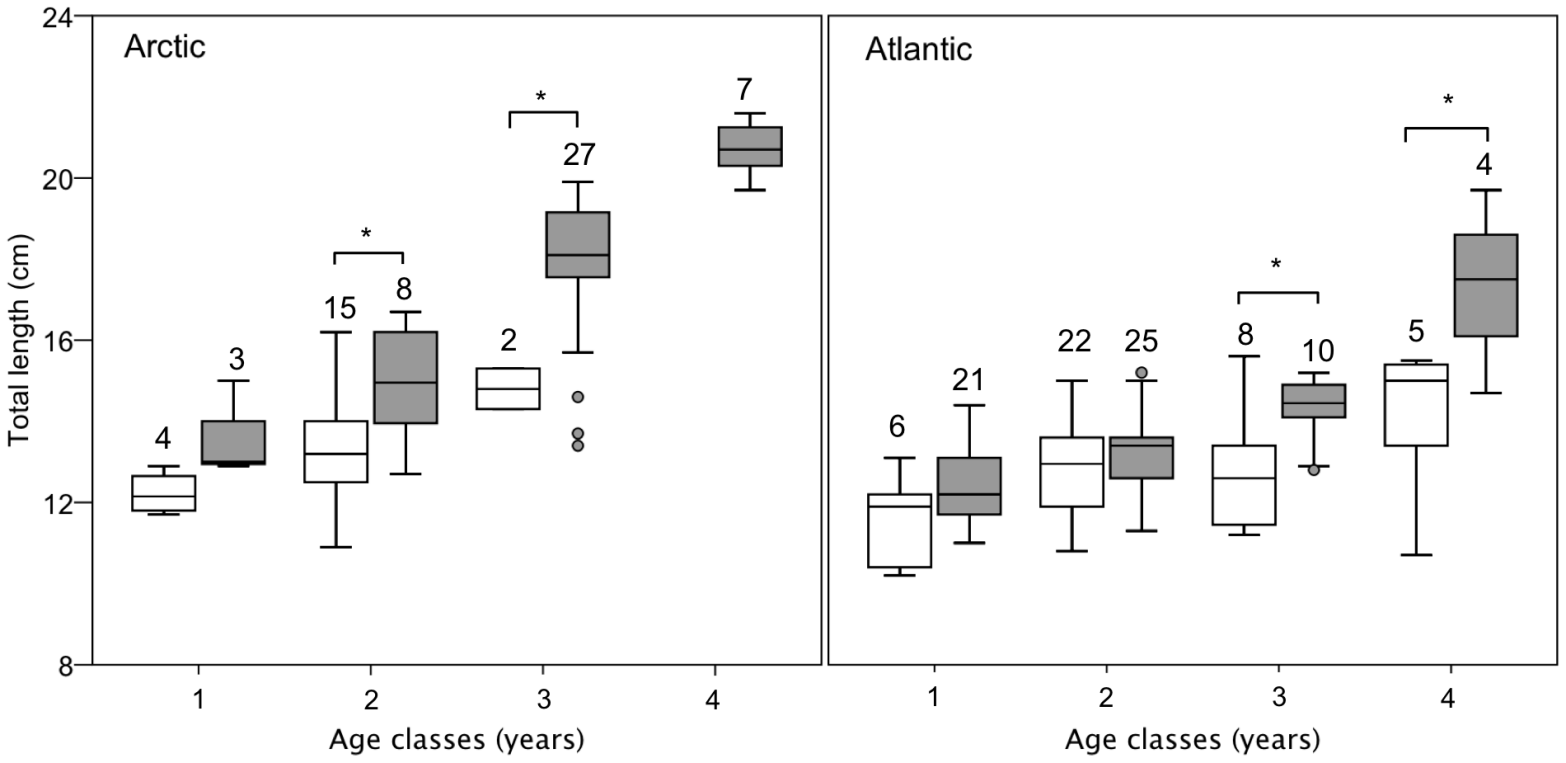
Total length (cm) of polar cod in relation to otolith based age (years) and maturity status. Mature (grey boxes) and immature (white boxes) individuals sampled in January 2011, 2012 and 2013 within the Arctic (n = 66) and Atlantic (n = 104) domains. For both domains, both genders were merged due to otherwise small sample size and because gender was not found to explain variability in length in the linear model operating on the larger dataset. Plots represent the median (line), 25%–75% percentiles (box), non-outlier range (wisker) and outliers (circle). Numbers above each box are n. Asterisks show significant (t-test without corrections for multiple comparisons, p<0.05) differences between mature and immature fish within domain and age-group.

For calculation of maturity data, we could only include the January samples (see above), reducing sample size to 170 individuals, 66 in the Arctic and 104 in the Atlantic. Instead of including maturity in the linear model we tested for differences in length between mature and immature fish within domain and age-group (T-tests without corrections for multiple comparisons, Figure 5). For mature fish we pooled males and females as gender did not contribute significantly in the larger linear model above.

Potential statistical differences in GSI between gender, size classes or domains (e.g. Table 2) were analysed using IBM SPSS statistics version 19.0. Requirements for normality and homogeneity of variances were not met, therefore the robust tests of equality of means (Welch-ANOVA) was used, followed by a multiple comparison test (Dunnett T3 test) to distinguish specific differences among groups. For all analyses the significance level was set to p≤0.05.

## Results and Discussion

The total dataset consists of 9165 specimens of wild caught polar cod, all of which are included in analyses on population structure (Table 1). In all other analyses, only a subset of the entire data contained the variables of interest, hence sample sizes varies and are smaller than for the population structure analysis (see Table S1). It is important to point out that we have taken the opportunity to gather all existing data from polar cod made available from scientific cruises around Svalbard during the last 4 years to provide a more holistic and general prospective on life history characteristics of polar cod. Due to this, the sampling design was often heterogenous and not appropriate to explain the mechanisms behind the observed trends. We do, however, highlight potential hypotheses for the observed patterns.

### Climatic domains and polar cod population structure

The two climatic domains of the present study were defined based on oceanographical characteristics as well as the diversity of potential prey and predators. The high Arctic archipelago of Svalbard is characterized by water masses of both Atlantic and Arctic origin (Figure 2) [30]. Rijpfjorden as well as Billefjorden [31] and the southern part of Hinlopen are predominantly influenced by Arctic water masses due to their location (Figure 2), and are therefore referred to as “Arctic-type” fjords [30]. In contrast, the fjords on the west coast of Svalbard (Bellsund, Isfjorden, Adventfjorden, Kongsfjorden and Krossfjorden) are largely dominated by warm water masses without formation of seasonal sea ice (referred to as ‘Atlantic type’ fjord [28], [32], Figure 2). It is important to note that in our approach, we do not consider if temperature is directly influencing the populations of polar cod (see also below). Rather, temperature is used as an indicator of differences in both the physical and biological habitat of the two domains.

The Atlantic domains are characterized by higher presence of potential predators on polar cod (Table 1), first of all the Atlantic cod (*Gadus morhua*) [33], [34]. Similarly, the composition of polar cod prey in the present study (Table 3) is markedly different between Atlantic and Arctic domains. Polar cod from the Arctic domain fed almost exclusively on the large Arctic amphipod *Themisto libellula*, while in the Atlantic domain, the diet was more diverse and included both mysids, cumaceans, polychaetes and fish (Table 3). The frequency of occurrence of *Themisto* spp. in the Atlantic domain was only 35%, of which more than two thirds were the Atlantic *T. abyssorum*. Similar observations have been made by previous authors [11], [35], but never before interpreted at a regional (domain) scale.

Polar cod from Arctic domains generally showed a broader size range and a higher abundance of large specimens (Table 1, Figure S3). In accordance with previous anecdotal observations from the western coast of Spitsbergen (Atlantic domains) [36] and Cheshskaya Bay and Novaya Zemlya (Arctic domain) [37], the polar cod length-at-age was higher in the Arctic compared to the Atlantic domain (Figure 4). In addition, previous studies from the American and Canadian Arctic [38], [39] have also reported differences in the size of polar cod between inshore and offshore areas. The differences in size were attributed to habitat differences, with inshore habitat providing favorable growing conditions (warmer coastal waters) as well as better protection against predation. Our analyses of body length variability suggested an increasing effect of domain with age and a starting point where young fish (age 1) did not differ between domains (Figure 4, Table S4). Furthermore, length-at-age did not depend on gender (Table S4). The difference in length-at-age between domains could not be explained by a shifted trade-off between growth in length versus body weight, as the relationship between total length and total weight was similar for both domains (Figure S1). The mechanisms resulting in a different length-at-age of polar cod between domains (Figure 4) are likely complex. A lower length-at-age, and earlier age-at-maturation (see discussion below) of polar cod from the Atlantic domain, can have several explanations including direct temperature effects on growth, higher predation risk, higher inter-specific species competition or density dependent effects of polar cod itself (such as intra-specific competition) [11], [19], [27], [40]. Of these four potential explanations, we regard the latter two to be of least importance. A previous study has suggested that the potential interspecific competition for food is limited between gadoid species of equal size [11]. Also, based on our density estimates we find it unlikely that density dependent effects are at play, even at the highest abundances reported herein for Isfjorden and Kongsfjorden (September 2013, Table 1) where mean abundance did not exceed 40 individuals km^−2^. Future studies should however apply acoustical methods for more precise density estimates. Finally, one of the most striking differences between the two domains, in terms of species composition is the general low numbers of large piscivorous fish predators in the Arctic domain ([11], [41] and Table 1). Furthermore, the diet of polar cod in the Atlantic domain may represent an energetic drop in foraging efficiency of adult fishes that may explain the smaller length-at-age found in polar cod from the Atlantic domain from age 2 (Figure 4). Early life stages of polar cod (up to 1), however, preferentially forage on *Calanus* spp [20]. Such a relationship between growth and availability of large Arctic zooplankton (mainly *T. libellula*) have previously been demonstrated for capelin in the Barents Sea [42]. Finally, although there are marked differences in temperature between the two domains (Figure 2), we have not analysed how much temperature may account for the observed patterns between domains. Temperature, however, plays a critical role, both directly on the physiology of organisms [40], but also in shaping the ecosystem structures such as predator/prey composition. In the present study, it is not possible to extract adequate temperature data in order to discriminate between direct effects of temperature on polar cod physiology and effects of temperature on ecosystem structure. Our spatial resolution only allows for two point measurements, not a continuous variable to which changes in polar cod populations could be related. In addition, with sampling being performed in different seasons, temperature snapshots at each sampling point (temporal and spatial) can not be used without making unsupported assumptions.

### Gender-specific reproductive strategy in the Arctic domain

In the Arctic domain, females have a longer life expectancy compared to males (Figure 3, Table S5), potentially reflecting a gender-specific trade-off between growth and reproduction. Indeed, the sex ratio distribution against the age/size classes was relatively even in the lower age classes (age 1 to 3), while the relative proportion of females increased with age, reaching 100% at age class 5. Such a skewed sex ratio has been anecdotally reported for polar cod from other true Arctic regions [38] and references therein, [43]–[45], but without being discussed nor analysed. This repeatedly observed sex-ratio pattern in Arctic domains therefore appears to be a pan-Arctic phenomenon. Life history theory predicts trade-offs between growth and reproduction, especially for a species with a positive relationship between size and fecundity [26]. Body size may also limit the amount of resources that can be stored and thus the number of eggs produced [46], adding to the potential benefits of large size for reproduction. Accordingly, we found both an increasing percentage of maturing individuals per size class as well as an increasing gonadosomatic index (GSI) with size (Table 2). Furthermore, mature individuals of the Arctic domain were larger than immature individuals across all age classes (Figure 5). Assuming that female fecundity in polar cod is limited by body size [44], this factor in addition to the longer life expectancy of females (Figure 3) may indicate that there is a selective pressure favouring individuals to delay reproduction and invest more energy in somatic growth at young age. Males, on the other hand, for which body size does not restrict fecundity as much as in females [47], seemed to invest more energy (e.g. higher GSI, Table 2; earlier maturation in the season [44]) in reproduction at a young age. Indeed, a higher proportion of sexually mature males was observed in the smaller size classes compared to females (Table 2). Although eggs are more energy rich than sperm, a higher energy investment of males compared to females may translate as the sum of the costs to produce and maintain larger sized gonads for a longer period of time, including e.g. reduced motility and higher predation risks. Reproduction for males, therefore, may come with increased mortality costs (e.g. [47]) which may explain the absence of older males (Figure 3).

Polar cod have previously been considered a semelparous species, but it has been demonstrated that they are capable of reproducing in two consecutive years while in captivity [20], [44]. Our study supports a more iteroparous life history due to the high percentage of mature individuals within each size class (Table 2). We hypothesise that males reproduce earlier in life to reduce risks of predation, but at the expense of a higher energy investment at a small size leading to higher post-spawning mortality. This trade-off further suggests a male reproductive strategy closer to semelparity than the iteroparous female's strategy. Iteroparity versus semelparity are outcomes of trade-offs between growth, fecundity and survival [26]. Semelparity is for instance suggested in cases where adult mortality is expected to be high [47], [48]. Moreover, the degree of semelparity within a species may differ between males and females as suggested for capelin (*Mallotus villosus*) in the Barents Sea [47], [49].

### Altered reproductive strategy in the Atlantic domain

The gender-specific patterns were less pronounced within the Atlantic domain, although the sex ratio was still skewed towards females in all age/size classes (Figure 3, Table S4). Importantly, the difference between the two domains regarding sex ratios of older fish, is most likely an effect of Atlantic fishes not growing as large as within the Arctic domain. In the Atlantic domain however, a larger proportion of the females matured within the smallest size class compared to the Arctic domain (Table 2), and the energy investment in terms of GSI was reduced in both sexes compared to the Arctic domain (Table 2). Together with the altered sex-ratio pattern, these differences suggest a different reproductive strategy compared to the Arctic domain. Polar cod from the Atlantic domain appear to allocate more energy to reproduction at a young age, at the cost of somatic growth, possibly as an adaptation to higher predation risk (Table 1). Changes in reproductive strategies across latitudes and environments have been reported in other fish species. The most common example is that of the riverine American shad (*Alosa sapidissma*) which shows increased iteroparity with increasing latitudes and decreasing environmental predictability [50]. Similarly, capelin also expresses different reproductive strategies in different spawning habitats; iteroparity in beach spawning versus semelparity in open sea spawning capelin [49]. These differences are also paralleled by other life history traits of the two capelin populations; body size, distance of spawning migration and age at first maturity and are believed to result from differences in environmental characteristics of the spawning habitats (e.g. physical forcing and predation pressure [49]).

There was a trend in all age classes of mature individuals being longer than immature, although with a stronger pattern within the Arctic domain (Figure 5). Also, immature specimens showed a consistent but non-significant trend of a larger hepatosomatic index (HSI) compared to mature specimens (Table S2). These data suggest that mature specimens used their hepatic energy storage for gonadal growth [51]. We thus hypothesize that individuals with a reduced fitness, characterized by a reduced length-at-age and lower energy reserves (reduced HSI), would skip reproduction in a given year. This is supported by a recent study [52] on the Northeast Arctic cod (*Gadus morhua*) where skipped spawning is common for individuals that fail to build up sufficient energy reserves for spawning.

Our fecundity estimates (Table 4) are within the ranges of previously published estimates [38], [39], [42], and suggest that polar cod from the Atlantic domain are at a disadvantage compared to the Arctic domain. At the individual level (Table 2) and within each size class, the estimated fecundity varied only by 22–28% between domains. However, assuming iteroparity for females, the expected lifetime fecundity of each female (sum of all eggs produced) was twice as high in the Arctic compared to the Atlantic domain (75000 and 39000 eggs per female, respectively). Even if assuming semelparity (average number of eggs produced across all size classes) for females, the larger size-at-age in the Arctic leads to a higher lifetime fecundity for Arctic than for Atlantic females (19000 and 13000 eggs per female, respectively). This suggests lower fitness for Atlantic domain- compared to Arctic domain polar cod. The factors that varied between domains included probability of maturing, size- and GSI-at-age. Hence, the earlier age-at-maturation of Atlantic domain polar cod resulted in similar fecundity between domains in the lower size classes, but did not seem to fully compensate their reduced size and GSI compared to the Arctic domain. The total estimated egg production in the Arctic domain (Rijpfjorden) catches were 5.3 and 3.3 million eggs in 2012 and 2013 respectively compared to between 0.1 and 0.6 million eggs for the Atlantic domain catches (Table 4). Seen from a population perspective, there is thus an order of magnitude higher number of eggs produced in the Arctic versus the Atlantic domain. The factors controlling this pattern include both a larger size-at-age and a higher number of mature females in the Arctic domain. However, another important aspect to consider is the potential advantage of an increased growth season for eggs and larvae in the Atlantic domains [21], [23].

**Table 4:** Population fecundity estimates.

	Arctic		Atlantic		
	Rijp		Adv	Isf	Kong
TL (cm)	2012	2013	2012	2013	2013
**Estimated amount of maturing females**					
]9–12]	21.1	1.4	4.2	12.6	2.5
]12–15]	177.2	119.2	17.4	33.2	5.5
]15–18]	17.1	154.3	2.6	8.6	3.2
]18–21]	20.3	34.0			
**Estimated total amount of eggs (10^3^)**					
]9–12]	163	10	23	70	14
]12–15]	2092	1407	160	305	50
]15–18]	331	2984	62	210	77
]18–21]	740	1243.			
**Total eggs in population (10^6^)**					
]9–21]	3.3	5.6	0.3	0.6	0.1

Estimation of the total amount of maturing females and eggs produced in the catch of five trawl hauls (January 2013, 2013) in the Arctic domain (Rijpfjorden) and Atlantic domain (Adventfjorden, Isfjorden and Kongsfjorden). The numbers were estimated based on data from Figure 3, Table 2 and the total polar cod population of each trawl haul (Fig S3).

### Consequences of climate change for polar cod and the ecosystem

We document that polar cod exhibit different reproductive and growth patterns between the Arctic and Atlantic domains. Although mechanistically not fully understood, our study suggests that a warmer climate will lead to changes in phenotypic traits, including earlier maturation, smaller size, increased investment in reproduction at early age, and in sum a reduced fecundity. These differences may be due to both phenotypic plasticity as well as differential selection acting on isolated populations. It is, however, at present not possible to fully discriminate between the two, and both explanations may to some degree be valid. However, previous studies have not been able to reveal a genetic isolation of populations between the Pechora Sea, Svalbard and Greenland [53]. As such, phenotypic plasticity may be the most important process. Importantly, however, regardless of the mechanisms at play, the observed differences in both population structure, gender balance and age-at-maturation are likely to have important ecological cascading effects, and are as such important to consider.

Representing a wide range of coastal habitats (open and silled fjords, straits and shelf regions on the western and northern side of Svalbard), and in line with previous studies from other parts of the Arctic (e.g. [38] and references therein, [45]), we argue that our observed patterns are relevant at a pan-Arctic scale. We therefore, hypothesize that a differential diet combined with increased predation on polar cod in the Atlantic domain may have caused the observed phenological differences. Although polar cod has been suggested as a central spawner, performing spawning migrations to key areas south of Svalbard and southeast of Novaya Zemlya [54], observations of both mature individuals (with GSI>20%) and young-of-the-year polar cod widely distributed in all fjords of Svalbard rather suggest that polar cod populations spawn locally [11]. Hence, polar cod is more likely to be affected by changes in the local environment than a species conducting large-scale spawning migrations. This hypothesis is further supported by Bouchard and Fortier [23], showing that water masses with differential temperatures and salinities may contribute to important phenological changes in the hatching season of polar cod larvae. We therefore hypothesise that climate change at the pan-Arctic scale may lead to similar patterns in polar cod populations, as those currently seen locally within Atlantic domains around Svalbard. These shifts seem to be a response to environmental forcing (e.g. predation pressure, prey availability and temperature) through changed trade-offs between somatic growth and reproduction among all size and age classes. Should polar cod be affected by climate change in this way, cascading effects to top predators are likely. Although mammalian predators may shift their preferences towards larger prey when available, Atlantic cod and other large temperate fish species are known to migrate seasonally, and are less available for non-migratory top predators such as the harbour seal [33], [55], [56] during winter and spring. During this time, such predators will be forced to shift diet back to polar cod. It is therefore likely that smaller sized polar cod will have significant impact on food web interactions and transfer of energy from the base of the food chain and up to top predators. Finally, a population fecundity reduced by one order of magnitude is likely to have significant implications for the global polar cod stock and further alter ecosystem structure and functioning.

In conclusion, we present unique evidence of a gender and domain specific reproduction strategy in polar cod, and how the predicted warming of the Arctic might alter these in the future. We argue that the differential predation pressure and prey availability, as well as a temperature difference between Arctic and Atlantic domains may play a substantial role in the observed reproductive patterns. Ultimately, we suggest that the findings from this study may provide a glimpse of what a future warmer Arctic may lead to. In a contiguous Arctic Ocean and shelf seas, a dramatic reduction in polar cod fecundity and size-at-maturation will have ecological cascading effects in the food web and play a significant role for future ecosystem services in Arctic areas affected by global warming.

## Supporting Information

Figure S1 **Relationship between total length (cm) and total weight (g) of polar cod.** Cubic regression line from the Arctic (n = 547, R^2^ = 0.97) and Atlantic (n = 577, R^2^ = 0.97) domains.
(TIF)

Figure S2 **Relationship between total length (cm) and fork length (cm) of polar cod (n = 1463).** Data includes specimens from November 2010 (Isf), September 2011 (Hin), January 2011 (Adv, Isf, Bell), April 2012 (Adv) and September 2012 (Bill, Hin, Kong, Rijp).
(TIF)

Figure S3 **Example of polar cod population size structure in January 2012 and January and September 2013.** Arctic domain Rijpfjorden (black continuous line) and Hinlopen (dot-dashed, Sept 2013) and in the Atlantic domain Adventfjorden (dotted line, in Jan 2012), Isfjorden (dotted line in Sept 2012 and 2013) and Kongsfjorden (dashed line). Numbers in brackets indicate total amount of polar cod in each trawl haul.
(TIF)

Table S1 **Overview of the sampling stations and number of specimens considered for each analyses.** Fish <10 cm in length were excluded, except for the population structure data (Table 1). Number of specimens between analyses can differ due to lack of adequate information for some specimens. For instance, January 2012 Rijpfjorden, otoliths were available for 73 specimens (Fig 3), but GSI was only present for 66 specimens (Table 2 and Fig 5).
(DOCX)

Table S2 **Hepatosomatic index (HSI%, mean ±SD) of polar cod.** Immature (I) and mature (M) polar cod were collected in January 2011, 2012 and 2013, from the Arctic (162) and Atlantic (104) domains. Numbers in bold and italics are significant differences (T-test) in means between immature and mature polar cod for a domain and size class (cm).
(DOCX)

Table S3 **Histological analysis of gonads of mature polar cod from January 2011 (Isfjorden) and 2012 (Rijpfjorden).** The Table shows mean occurrence of oocyte stages (%) based on oocyte counts (n = 254 oocyte counts), mean diameter (mm) and weight (ug) of each oocyte stage: oogonia (Oo), previtellogenesis (PVit), cortical alveoli (CA), lipid inclusions formation (LIF), vitellogenesis (Vit), maturation (Mat), post-ovulatory follicles (POF), atretic oocytes (AO). There were no statistically significant differences in egg size (t-test, p<0.05) or maturation stages between domains and years.
(DOCX)

Table S4 **Summary output from a linear model for polar cod total length, explained by age, climatic domain as well as the interaction between age and domain.** This model explained 65% of the variability in polar cod body length. Gender and all other interaction terms were omitted as explanatory variables after model simplification based on stepwise deletion of non-significant terms. P-values were smaller than 0.005 for all variables in the table. n = 281. Parameter estimate for intercept and slope in the table is for the Arctic domain. Intercept and slope for the Atlantic is obtained by adding the estimates for domain and the interaction term.
(DOCX)

Table S5 **Percentage female polar cod per age class (total n per age class) from the Arctic domain stations (upper panel) and Atlantic domain stations (lower panel).** Age classes are derived from the linear regression presented in Fig 3. Fish <10 cm in length were excluded. Bill: Billefjorden, Hin: Hinlopen, Rijp: Rijpfjorden Isf: Isfjorden, Adv: Adventfjorden, Kong: Kongsfjorden, Kross: Krossfjorden, Bell: Bellsund.
(DOCX)
