# Supplementary figures and images for: Gender Specific Reproductive Strategies of an Arctic Key Species (Boreogadus saida) and Implications of Climate Change
Source: PLoS One. 2014 May 28;9(5):e98452. doi: 10.1371/journal.pone.0098452 (PMC4037215; doi:10.1371/journal.pone.0098452)

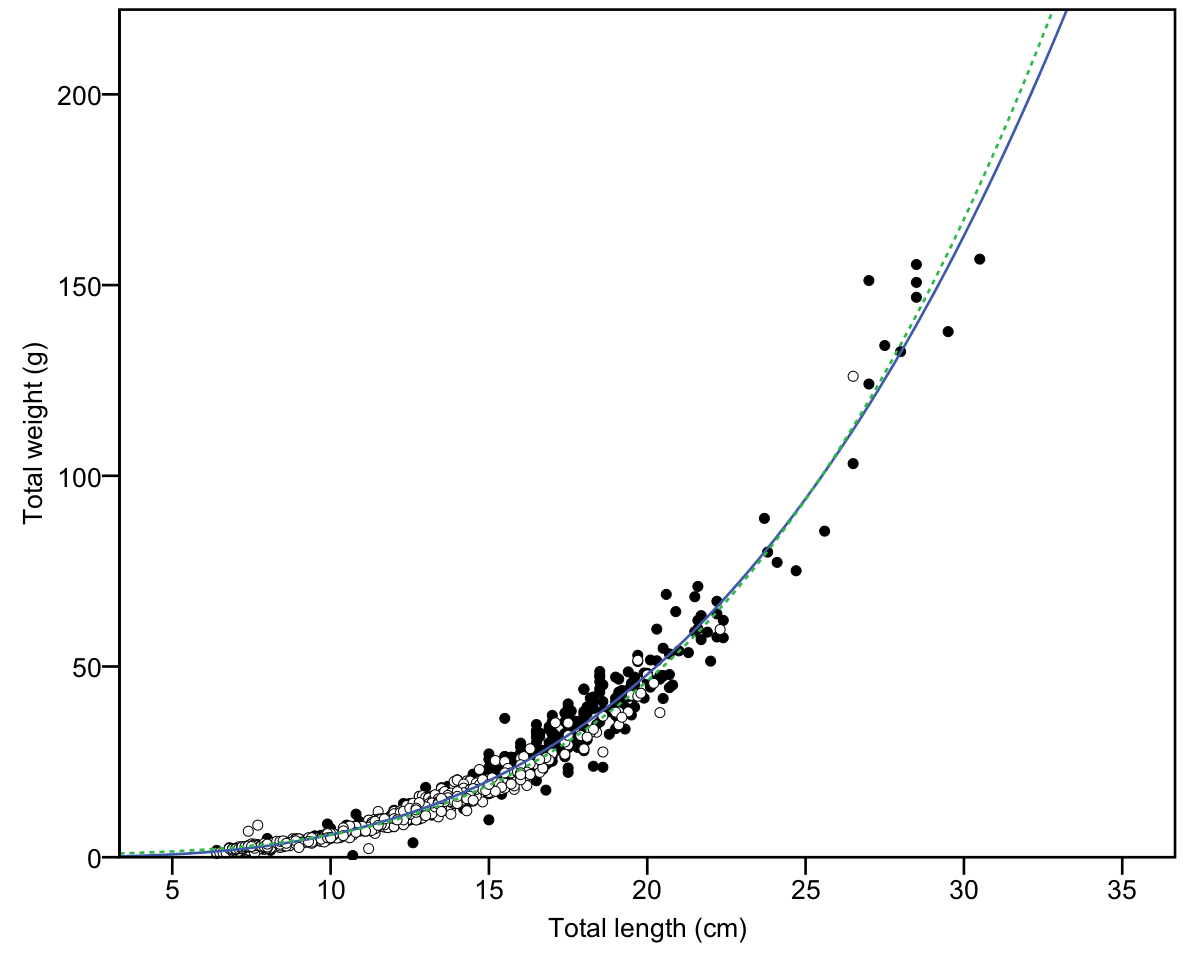

Supplement: Figure S1 — Relationship between total length (cm) and total weight (g) of polar cod. Cubic regression line from the Arctic (n = 547, R2 = 0.97) and Atlantic (n = 577, R2 = 0.97) domains. (TIF) [file pone.0098452.s001.tif]

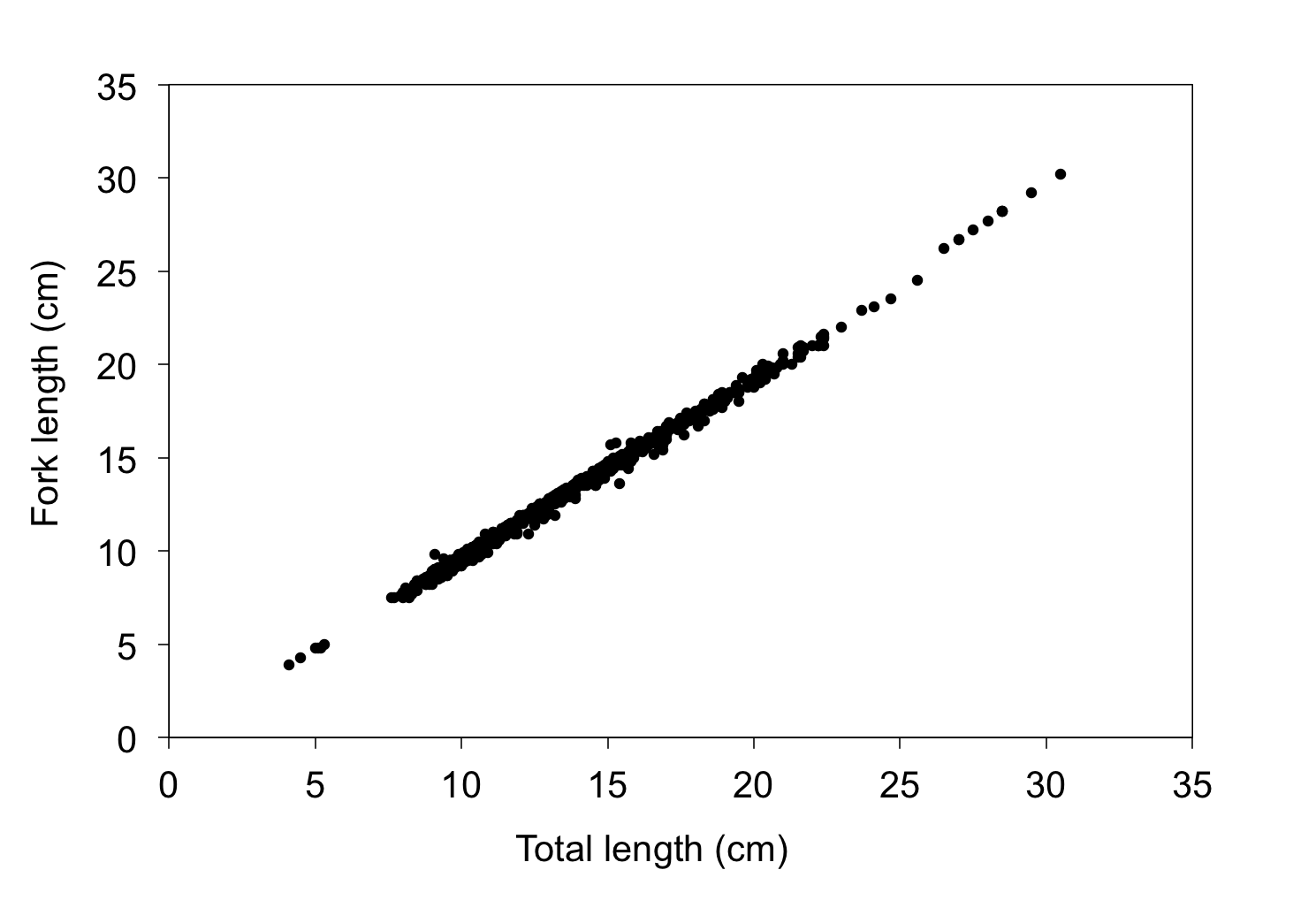

Supplement: Figure S2 — Relationship between total length (cm) and fork length (cm) of polar cod (n = 1463). Data includes specimens from November 2010 (Isf), September 2011 (Hin), January 2011 (Adv, Isf, Bell), April 2012 (Adv) and September 2012 (Bill, Hin, Kong, Rijp). (TIF) [file pone.0098452.s002.tif]

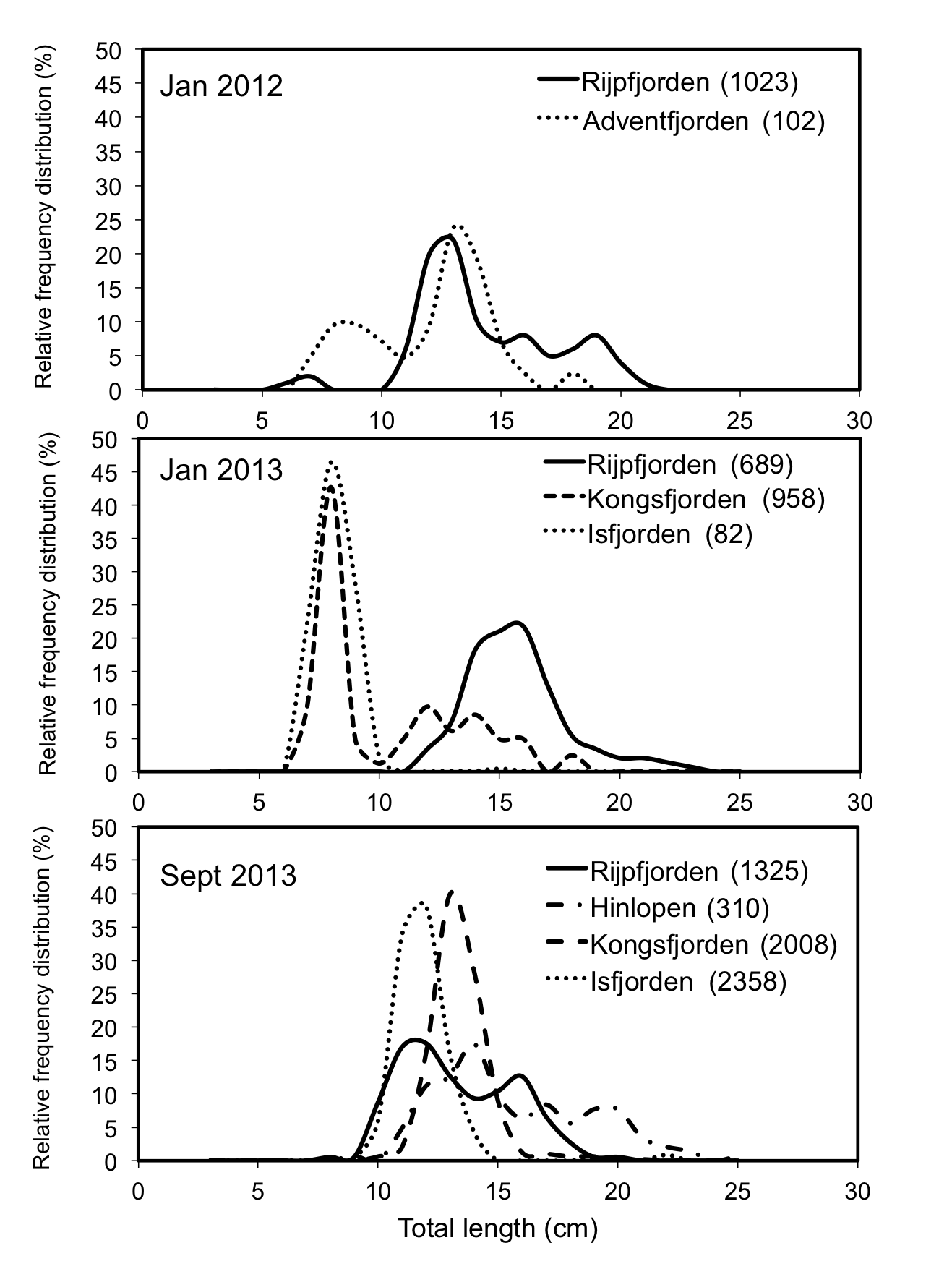

Supplement: Figure S3 — Example of polar cod population size structure in January 2012 and January and September 2013. Arctic domain Rijpfjorden (black continuous line) and Hinlopen (dot-dashed, Sept 2013) and in the Atlantic domain Adventfjorden (dotted line, in Jan 2012), Isfjorden (dotted line in Sept 2012 and 2013) and Kongsfjorden (dashed line). Numbers in brackets indicate total amount of polar cod in each trawl haul. (TIF) [file pone.0098452.s003.tif]
