# Supplementary material for: Gender Specific Reproductive Strategies of an Arctic Key Species (Boreogadus saida) and Implications of Climate Change
Source: PLoS One. 2014 May 28;9(5):e98452. doi: 10.1371/journal.pone.0098452 (PMC4037215; doi:10.1371/journal.pone.0098452)
Supplement: Table S1 — Overview of the sampling stations and number of specimens considered for each analyses. Fish <10 cm in length were excluded, except for the population structure data (Table 1). Number of specimens between analyses can differ due to lack of adequate information for some specimens. For instance, January 2012 Rijpfjorden, otoliths were available for 73 specimens (Fig 3), but GSI was only present for 66 specimens (Table 2 and Fig 5). (DOCX) [file pone.0098452.s004.docx]

**Table S1. Overview of the sampling stations and number of specimens considered for each analyses.**

Fish <10cm in length were excluded, except for the population structure data (Table 1). Number of specimens between analyses can differ due to lack of adequate information for some specimens. For instance, January 2012 Rijpfjorden, otoliths were available for 73 specimens (Fig 3), but GSI was only present for 66 specimens (Table 2 and Fig 5).

| Month | Station | Climatic domains | Table 1 | Table 2 | Table 3 | Fig 3 | Fig 4 | Fig 5 |
| --- | --- | --- | --- | --- | --- | --- | --- | --- |
| November 2010 | Isfjorden | Atlantic |  |  |  | 37 | 37 |  |
|  |  |  |  |  |  |  |  |  |
| January 2011 | Isfjorden | Atlantic |  | 30 |  | 30 | 30 | 30 |
|  | Adventfjorden |  |  | 11 |  | 11 | 11 | 11 |
|  | Bellsund |  |  | 23 |  | 23 | 23 | 23 |
|  |  |  |  |  |  |  |  |  |
| September 2011 | Kongsfjorden | Atlantic |  |  |  |  | 87 |  |
|  | Billefjorden | Arctic |  |  |  | 22 | 94 |  |
|  | Rijpfjorden |  |  |  |  |  | 71 |  |
|  | Hinlopen |  |  |  |  |  | 40 |  |
|  |  |  |  |  |  |  |  |  |
| January 2012 | Adventfjorden | Atlantic | 102 |  |  |  | 25 |  |
|  | Rijpfjorden | Arctic | 1023 | 66 |  | 73 | 206 | 66 |
|  |  |  |  |  |  |  |  |  |
| April 2012 | Adventfjorden | Atlantic |  |  |  | 58 | 149 |  |
|  |  |  |  |  |  |  |  |  |
| September 2012 | Kongsfjorden | Atlantic |  |  |  |  | 65 |  |
|  | Billefjorden | Arctic |  |  |  |  | 193 |  |
|  | Rijpfjorden |  |  |  |  |  | 588 |  |
|  | Hinlopen |  |  |  |  |  | 176 |  |
|  |  |  |  |  |  |  |  |  |
| January 2013 | Kongsfjorden | Atlantic | 82 | 26 |  | 26 | 34 | 26 |
|  | Krossfjorden |  | 310 | 11 |  | 8 | 18 | 8 |
|  | Isfjorden |  | 958 | 6 |  | 6 | 8 | 6 |
|  | Rijpfjorden | Arctic | 689 | 141 |  |  | 147 |  |
|  |  |  |  |  |  |  |  |  |
| September 2013 | Kongsfjorden | Atlantic | 2008 |  | 157 |  | 98 |  |
|  | Krossfjorden |  |  |  |  |  | 18 |  |
|  | Isfjorden |  | 2358 |  |  |  | 125 |  |
|  | Rijpfjorden | Arctic | 1325 |  | 298 |  | 204 |  |
|  | Hinlopen |  | 310 |  |  |  | 94 |  |
| Total |  |  | 9165 | 314 | 455 | 296 | 2541 | 170 |
