# Supplementary material for: Gender Specific Reproductive Strategies of an Arctic Key Species (Boreogadus saida) and Implications of Climate Change
Source: PLoS One. 2014 May 28;9(5):e98452. doi: 10.1371/journal.pone.0098452 (PMC4037215; doi:10.1371/journal.pone.0098452)
Supplement: Table S2 — Hepatosomatic index (HSI%, mean ±SD) of polar cod. Immature (I) and mature (M) polar cod were collected in January 2011, 2012 and 2013, from the Arctic (162) and Atlantic (104) domains. Numbers in bold and italics are significant differences (T-test) in means between immature and mature polar cod for a domain and size class (cm). (DOCX) [file pone.0098452.s005.docx]

**Table S2. Hepatosomatic index (HSI %, mean ± SD) of polar cod.**

Immature (I) and mature (M) polar cod were collected in January 2011, 2012 and 2013, from the Arctic (162) and Atlantic (104) domains. Numbers in bold and italics are significant differences (T-test) in means between immature and mature polar cod for a domain and size class (cm).

|  | Arctic | |  | Atlantic | |
| --- | --- | --- | --- | --- | --- |
| TL (cm) | I | M |  | I | M |
| ]9-12] | ***7.8 ± 3.1*** | ***4.0 ± 2.0*** |  | 7.3 ± 3.0 | 6.5 ± 3.2 |
| ]12-15] | 6.2 ± 2.8 | 5.7 ± 2.4 |  | 7.2 ± 2.5 | 6.3 ± 2.8 |
| ]15-18] |  | 5.4 ± 2.8 |  |  | 5.0 ± 2.7 |
| ]18-21] |  | 6.7 ± 2.9 |  |  |  |
