# Supplementary material for: Gender Specific Reproductive Strategies of an Arctic Key Species (Boreogadus saida) and Implications of Climate Change
Source: PLoS One. 2014 May 28;9(5):e98452. doi: 10.1371/journal.pone.0098452 (PMC4037215; doi:10.1371/journal.pone.0098452)
Supplement: Table S3 — Histological analysis of gonads of mature polar cod from January 2011 (Isfjorden) and 2012 (Rijpfjorden). The Table shows mean occurrence of oocyte stages (%) based on oocyte counts (n = 254 oocyte counts), mean diameter (mm) and weight (ug) of each oocyte stage: oogonia (Oo), previtellogenesis (PVit), cortical alveoli (CA), lipid inclusions formation (LIF), vitellogenesis (Vit), maturation (Mat), post-ovulatory follicles (POF), atretic oocytes (AO). There were no statistically significant differences in egg size (t-test, p<0.05) or maturation stages between domains and years. (DOCX) [file pone.0098452.s006.docx]

**Table S3. Histological analysis of gonads of mature polar cod from January 2011 (Isfjorden) and 2012 (Rijpfjorden).**

The Table shows mean occurrence of oocyte stages (%) based on oocyte counts (n=254 oocyte counts), mean diameter (mm) and weight (ug) of each oocyte stage: oogonia (Oo), previtellogenesis (PVit), cortical alveoli (CA), lipid inclusions formation (LIF), vitellogenesis (Vit), maturation (Mat), post-ovulatory follicles (POF), atretic oocytes (AO). There were no statistically significant differences in egg size (t-test, p<0.05) or maturation stages between domains and years.

|  | Oogenesis stages | | | | | | | AO |
| --- | --- | --- | --- | --- | --- | --- | --- | --- |
|  | Oo | Pvit | CA | LIF | Vit | Mat | POF |  |
| % mean occurrence | 24 | 50 | 0 | 0 | 24 | 0 | 0 | 2 |
| Mean diameter (mm) | 0.028 | 0.109 | 0 | 0 | 0.606 | 0 | 0 | 0.091 |
| Oocyte weight (ug)* | 0.01 | 0.68 | 0 | 0 | 116.47 | 0 | 0 | 0.39 |
| Percentage weight of 100 eggs | 0.01 | 1.2 | 0 | 0 | 98.8 | 0 | 0 | 0.03 |
