# Supplementary material for: Gender Specific Reproductive Strategies of an Arctic Key Species (Boreogadus saida) and Implications of Climate Change
Source: PLoS One. 2014 May 28;9(5):e98452. doi: 10.1371/journal.pone.0098452 (PMC4037215; doi:10.1371/journal.pone.0098452)
Supplement: Table S4 — Summary output from a linear model for polar cod total length, explained by age, climatic domain as well as the interaction between age and domain. This model explained 65% of the variability in polar cod body length. Gender and all other interaction terms were omitted as explanatory variables after model simplification based on stepwise deletion of non-significant terms. P-values were smaller than 0.005 for all variables in the table. n = 281. Parameter estimate for intercept and slope in the table is for the Arctic domain. Intercept and slope for the Atlantic is obtained by adding the estimates for domain and the interaction term. (DOCX) [file pone.0098452.s007.docx]

**Table S4. Summary output from a linear model for polar cod total length, explained by age, climatic domain as well as the interaction between age and domain.** This model explained 65% of the variability in polar cod body length. Gender and all other interaction terms were omitted as explanatory variables after model simplification based on stepwise deletion of non-significant terms. P-values were smaller than 0.005 for all variables in the table. n = 281. Parameter estimate for intercept and slope in the table is for the Arctic domain. Intercept and slope for the Atlantic is obtained by adding the estimates for domain and the interaction term.

|  | Estimate | Std. Error | t value |
| --- | --- | --- | --- |
| (Intercept) | 8.898 | 0.495 | 17.97 |
| Age | 2.710 | 0.182 | 14.89 |
| Domain (Atlantic) | 1.858 | 0.571 | 3.25 |
| Age:Domain (Atlantic) | -1.628 | 0.225 | -7.23 |
