# Supplementary material for: Gender Specific Reproductive Strategies of an Arctic Key Species (Boreogadus saida) and Implications of Climate Change
Source: PLoS One. 2014 May 28;9(5):e98452. doi: 10.1371/journal.pone.0098452 (PMC4037215; doi:10.1371/journal.pone.0098452)
Supplement: Table S5 — Percentage female polar cod per age class (total n per age class) from the Arctic domain stations (upper panel) and Atlantic domain stations (lower panel). Age classes are derived from the linear regression presented in Fig 3. Fish <10 cm in length were excluded. Bill: Billefjorden, Hin: Hinlopen, Rijp: Rijpfjorden Isf: Isfjorden, Adv: Adventfjorden, Kong: Kongsfjorden, Kross: Krossfjorden, Bell: Bellsund. (DOCX) [file pone.0098452.s008.docx]

**Table S5. Percentage female polar cod per age class (total n per age class) from the Arctic domain stations (upper panel) and Atlantic domain stations (lower panel).**

Age classes are derived from the linear regression presented in Fig 3. Fish <10 cm in length were excluded. Bill: Billefjorden, Hin: Hinlopen, Rijp: Rijpfjorden Isf: Isfjorden, Adv: Adventfjorden, Kong: Kongsfjorden, Kross: Krossfjorden, Bell: Bellsund.

| Arctic domain | Bill | Bill | Hin | Hin | Hin | Rijp | Rijp | Rijp | Rijp | Rijp |  |  |
| --- | --- | --- | --- | --- | --- | --- | --- | --- | --- | --- | --- | --- |
| Age classes (years) | Sept 2011 | Sept 2012 | Sept 2011 | Sept 2012 | Sept 2013 | Sept 2011 | Sept 2012 | Jan 2012 | Jan 2013 | Sept 2013 |  |  |
| 0 | 33 (6) | 41 (168) |  | 55 (20) |  | 42 (12) | 39 (180) | 52 (42) | 40 (5) | 0 (11) |  |  |
| 1 | 45 (20) | 33 (18) |  | 50 (28) | 0 (1) | 67 (15) | 44 (190) | 54 (76) | 36 (39) | 40 (5) |  |  |
| 2 | 47 (36) | 33 (3) | 0 (1) | 47 (38) | 58 (24) | 43 (21) | 52 (172) | 50 (32) | 61 (74) | 56 (131) |  |  |
| 3 | 61 (28) | 25 (4) | 67 (18) | 59 (61) | 71 (52) | 38 (21) | 34 (35) | 70 (43) | 57 (23) | 79 (56) |  |  |
| 4 | 67 (3) |  | 73 (11) | 69 (26) | 75 (16) | 50 (2) | 50 (10) | 54 (13) | 67 (6) | 100 (1) |  |  |
| 5 | 100 (1) |  | 100 (5) | 100 (3) | 100 (1) |  | 100 (1) |  |  |  |  |  |
| 6 |  |  | 100 (5) |  |  |  |  |  |  |  |  |  |
| 7 |  |  |  |  |  |  |  |  |  |  |  |  |
| Total n | 94 | 193 | 40 | 176 | 94 | 71 | 588 | 206 | 147 | 204 |  |  |
|  |  |  |  |  |  |  |  |  |  |  |  |  |
| Atlantic domain | Isfj | Isfj | Isfj | Adv | Adv | Kong | Kong | Kong | Kong | Kross | Kross | Bell |
| Age classes (years) | Nov 2010 | Jan 2011 | Sept 2013 | April 2012 | Jan 2012 | Sept 2011 | Sept 2012 | Jan 2013 | Sept 2013 | Jan 2013 | Sept 2013 | Jan 2011 |
| 0 | 25 (4) | 38 (8) | 52 (29) | 47 (47) | 17 (6) | 56 (39) | 50 (4) | 55 (11) |  | 17 (6) |  | 75 (4) |
| 1 | 43 (7) | 53 (17) | 53 (15) | 44 (18) | 18 (11) | 63 (16) | 42 (19) | 50 (4) |  |  |  | 83 (6) |
| 2 | 56 (18) | 69 (13) | 0 (2) | 83 (29) | 60 (5) | 50 (4) | 27 (11) | 44 (9) |  | 50 (2) |  | 45 (11) |
| 3 | 50 (2) | 100 (3) | 0 (3) | 78 (32) | 0 (1) | 80 (5) | 100 (7) | 33 (6) | 80 (15) | 33 (3) |  | 50 (2) |
| 4 | 25 (4) |  | 75 (4) | 59 (17) |  | 56 (9) | 70 (10) | 50 (2) | 74 (27) | 100 (1) | 0 (1) |  |
| 5 | 0 (2) |  | 65 (20) | 67 (3) | 0 (1) | 25 (4) | 91 (11) | 50 (2) | 87 (15) |  | 17 (6) |  |
| 6 |  |  | 75 (20) | 0 (1) | 0 (1) | 63 (8) | 0 (2) |  | 87 (15) | 100 (2) | 60 (5) |  |
| 7 |  |  | 88 (32) | 100 (2) |  | 50 (2) | 100 (1) |  | 88 (26) | 50 (2) | 83 (6) |  |
| Total n | 37 | 41 | 125 | 149 | 25 | 87 | 65 | 34 | 98 | 16 | 18 | 23 |
